# Fresh Umbilical Cord Blood—A Source of Multipotent Stem Cells, Collection, Banking, Cryopreservation, and Ethical Concerns

**DOI:** 10.3390/life13091794

**Published:** 2023-08-23

**Authors:** Seeta Devi, Anupkumar M. Bongale, Minyechil Alehegn Tefera, Prashant Dixit, Prasad Bhanap

**Affiliations:** 1 Department of Obstetrics and Gynecological Nursing, Symbiosis College of Nursing, Symbiosis International (Deemed University), Lavale, Pune 412 115, Maharashtra, India; sitadevi@scon.edu.in; 2 Department of Artificial Intelligence and Machine Learning, Symbiosis Institute of Technology, Symbiosis International (Deemed University), Lavale, Pune 412 115, Maharashtra, India; 3 Department of Information Technology, Mizan Tepi University, Tepi 121, Ethiopia; 4 Howard Newborn Centre, Malad 400064, Mumbai, India; 5 HoD OBG Department, Symbiosis Medical College for Women (SMCW), Symbiosis International (Deemed University), Lavale, Pune 412 115, Maharashtra, India

**Keywords:** umbilical cord blood, mesenchymal stem cells, collection, banking, cryopreservation, ethical issues

## Abstract

Umbilical cord *blood* (UCB) is a rich source of hematopoietic cells that can be used to replace bone marrow components. Many blood disorders and systemic illnesses are increasingly being treated with stem cells as regenerative medical therapy. Presently, collected blood has been stored in either public or private banks for allogenic or autologous transplantation. Using a specific keyword, we used the English language to search for relevant articles in SCOPUS and PubMed databases over time frame. According to our review, Asian countries are increasingly using UCB preservation for future use as regenerative medicine, and existing studies indicate that this trend will continue. This recent literature review explains the methodology of UCB collection, banking, and cryopreservation for future clinical use. Between 2010 and 2022, 10,054 UCB stem cell samples were effectively cryopreserved. Furthermore, we have discussed using Mesenchymal Stem Cells (MSCs) as transplant medicine, and its clinical applications. It is essential for healthcare personnel, particularly those working in labor rooms, to comprehend the protocols for collecting, transporting, and storing UCB. This review aims to provide a glimpse of the details about the UCB collection and banking processes, its benefits, and the use of UCB-derived stem cells in clinical practice, as well as the ethical concerns associated with UCB, all of which are important for healthcare professionals, particularly those working in maternity wards; namely, the obstetrician, neonatologist, and anyone involved in perinatal care. This article also highlights the practical and ethical concerns associated with private UCB banks, and the existence of public banks. UCB may continue to grow to assist healthcare teams worldwide in treating various metabolic, hematological, and immunodeficiency disorders.

## 1. Introduction

Before delving into umbilical cord blood banking, it is imperative to recognize that stem cells are components extracted from human blood, bone marrow, body tissues, skeletal muscles, and embryos [1]. Stem cells replenish themselves by dividing and producing similar daughter cells. Stem cells have the ability to withstand the process of differentiation into specific offspring cells [2]. When stem cells divide asymmetrically, they produce one stem and one non-stem cell; the daughter non-stem cell can differentiate into a more specialized one, but the daughter stem cell regains “stemness” properties [3]. Stem cells differentiated into tissues derived from germinal layers such as ectoderm, endoderm, and mesoderm are “pluripotent”. The inner cell mass is the source of embryonic cells that are suitable examples of pluripotent stem cells [4].

Three types of stem cells are commonly found, namely *embryonic*, *adult*, and *UCB*. Embryonic Stem Cells (ESCs) are commonly used in clinical trials, and specialized cells produced from ESCs are deployed in replacing the damaged cells in several degenerative disease conditions; however, this is still under research. It is challenging to understand the past and potential benefits for patients of human embryonic stem cells, adult stem cells, and umbilical cord blood stem cells. In some studies, authors have suggested that specialized neurons could be derived from embryonic stem cells and transplanted into the brains of Parkinson’s disease patients [5,6]. Hopefully, there will be more successful human trials in the future, enabling the production of cells as well as organs from embryonic or induced pluripotent stem cells. This will encourage clinicians to consider treatments in the future for an increasing number of disease conditions with the cells generated from ESCs [7]. Most adult stem cells are found in the bone marrow of the human body. Over the years, bone stem cells have been used to treat various hematological and other systemic disorders. As per the current study’s reports, the employment of stem cells has effective and swift work in the bone regenerative process [8].

In the current article, the authors proposed to review the past and recent studies and clinical trials conducted using UCB stem cells. This paper also reviews the existence of various public and private banks available for the storage and cryopreservation of the UCB, and its practical and ethical implications. Umbilical Cord Blood (UCB) stem cells are traditionally thought to be an unwanted product of the birthing process, but they are one of the most well acknowledged sources of hematopoietic stem cells/hematopoietic progenitor cells (HSCs/HPCs), comparable to that present in the bone marrow and peripheral blood [9].

Immunological disorders, hematological conditions, malignancies, and congenital metabolic disorders are the most common disease conditions that are treated with stem cells. Stem cells are also used to treat cancers such as lymphoma and leukemia [10]. UCB stem cells treat a variety of hematological and metabolic disorders, including sickle cell, Fanconi’s anemia, and adrenoleukodystrophy [11]. Since 1989, umbilical cord stem cells have been used successfully for hematopoietic cell transplantation (HCT) [12]. Approximately 40,000 umbilical cord blood transplants are produced worldwide for the treatment of various cancerous and non-cancerous disorders. Furthermore, public banks are currently releasing approximately 30 times more UCB units than before [10,13,14]. The results of the UCB transplants are significantly better than those of adult or embryonic stem cells when used as a graft source in blood cancers [15,16,17,18]. Immunocompromised illnesses, bone marrow syndromes, and blood conditions, including sickle cell disease and thalassemia, remain to benefit from treatment with umbilical cord stem cells [19]. Clinical trials at www.clinicaltrials.gov provide evidence that UCB can be used successfully to treat a wide range of disease conditions.

It is essential for the healthcare professional who will be collecting the blood, to be well versed with the state and local laws pertaining to providing information and obtaining consent from the donors [20]. The UCB specimens are transferred either to the banks or laboratories, and are tracked by Laboratory Information Management Systems (LIMS) [21]. The introduction of UCB banking ensures cord stem cells’ safe and immediate availability to treat the aforementioned disorders. The Aids Clinical Trials Group (ACTG) has developed standard operating procedures for the collection of umbilical cord blood while adhering to biohazard protocols. This involves collecting the blood from the placenta or umbilical cord immediately after delivery, before the blood clotting mechanism is triggered. Strict aseptic precautions are taken during the collection process, and the blood is then collected in a 100 mL vacutainer and labeled accordingly [21].

The first UCB bank opened in 1993, and approximately five million UCB units are now stored worldwide. Private banks hold approximately 4 million UCB units, while government banks hold 800,000 UCB units [14]. The biological structures of UCB units can remain stable for HSCs and hematopoietic progenitor cells (HPCs) for more than twenty years under safe cryopreservation and appropriate conditions [22]. These days, private UCB banks are active in advertising and provide impetus to the donors to donate UCB in the form of biological insurance against anticipated future disorders. Consequently, donations of UCB samples are more common in private banks than in the public. A recent study conducted in Japan stated that approximately 34.4% and 6.1% of UCB samples were preserved in private and public banks, respectively [8]. To maintain their high standards, several organizations, including the National Telephone Donation Program, NetCord, and the Mobile Medical Authorization Framework use well-known monitoring strategies for collecting, dispensing, and maintaining UCB components [23].

In an antenatal context, women and their relatives are required to know about UCB donation and its potential uses, as it can increase awareness and the likelihood of donation, leading to more cord blood units for transplantation. Educating them can help treat a range of conditions, including those with a family history of certain diseases. Furthermore, front-line healthcare professionals working with antenatal and intranatal women must have an adequate awareness of cord blood stem cells, including their use, collection, and storage. Accurate information from these professionals increases awareness and informed decision-making about cord blood donation and storage, and ensures successful transplantation in the future. The review paper provides comprehensive information on the new evolution of umbilical cord blood stem cells, allowing healthcare professionals to gain a complete understanding of this topic. This article will serve as a one-stop shop for information about UCB stem cells, with quick updates on new developments.

UCB has been utilized as a means for regenerative cell therapy and immune modulation. As a result, the gathering and storage of cells derived from UCB has gained widespread popularity. However, there are issues regarding the expense and benefits associated with UCB banking, as well as the ethical and legal ramifications that arise, and its limited usage.

The drawbacks of UCB stem cells usage are as follows; expertise is required for the collection, storage, and processing of UCB. Stem cells derived from umbilical cord blood may not be beneficial for the donor, in the treatment of genetic disorders and cancer in the same person (known as an autologous transplant). This is because the stored cord blood will contain the same genetic variation or abnormal cells that caused the disease in the first place. Presently, there is no proof to suggest that autologous cord blood samples can be utilized in regenerative medicine.

The quantity of stem cells obtained in less than 50 to 200 mL of cord blood is insufficient for the treatment of adults, as it would only meet the needs of a child weighing 10 kg [24]. Just 8–10% of units have an adequate volume for adult use. Furthermore, the slow engraftment rates and high expenses are additional concerns [25].

There are several reasons to argue against private cord blood banking. Firstly, the likelihood that a person will require an autologous transfusion (using their own cord blood) ranges from 1 in 400 to 1 in 200,000. Secondly, there is no information available on the long-term durability of cord blood cells. Additionally, storing cord blood is costly, and the amount of blood collected may not be adequate for use in older children or adults [24].

The rest of the article is structured as follows: Section 2 provides a brief bibliometric analysis to understand the significance of the proposed review. Section 3 explains the use of UCB as a regenerative medicine, while Section 4 outlines the process of UCB collection and cryopreservation. Public and private UCB banking is discussed in Section 5, followed by a section on ethical considerations in UCB banking in Section 6. Finally, Section 7 provides a detailed article discussion and this is concluded in Section 8.

## 2. Brief Bibliometric Review

The current survey article is about the recent trends in the applications of UCB Stem Cell transplantation in several diseases arising due to hematopoietic stem cells and MSCs in regenerative therapies. To better understand the research trends in the proposed area of interest, a brief bibliometric analysis of the existing survey articles is presented in this section. Bibliometric analysis helps us to study the cited articles, research interaction, author affiliation, and funding information from the research article courses, popular journals, etc. To understand essential keywords for the current study, bibliometric indicators such as Annual Scientific Production, Country Scientific Production, and most globally cited survey articles pertinent to the subject area were used [26]. The bibliometric analysis has helped to set up the objectives of the proposed survey article.

### 2.1. Data Collection

**Figure 1:**
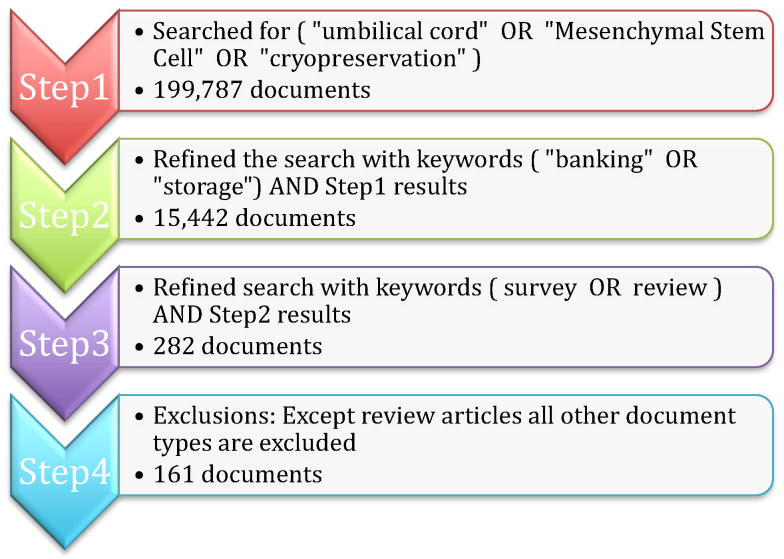
Data Collection Procedure.

The purpose of the bibliometric analysis is to understand the existing survey articles related to umbilical cord blood. The Scopus repository is one of the largest collections of citations and abstracts of peer-reviewed literature; hence, Scopus is considered for data collection for the bibliometric study. The steps shown in Figure 1 are followed for finalizing the search query and document collection process (the search query was executed in Scopus on 28 April 2023). All of the steps are explained below:

- **Step 1:** Initially, the search is carried out based on important keywords such as (“umbilical cord” OR “Mesenchymal Stem Cell” OR “cryopreservation”) in the title, abstract, and keywords of the Scopus repository. The search resulted in 199,787 research articles.
- **Step 2:** The search is further narrowed by considering additional keywords ( “banking” OR “storage”), along with the results of Step 1, 15,442 documents are obtained.
- **Step 3:** Since the study is focused only on survey articles, the search is further refined with the keywords ( survey OR review ) AND *Step 2* results to obtain 282 articles.
- **Step 4:** All of the articles that belong to the research experiments, book chapters, and conference proceedings are excluded to obtain the final 161 review research articles.

### 2.2. Bibliometric Indictors

**Figure 2:**
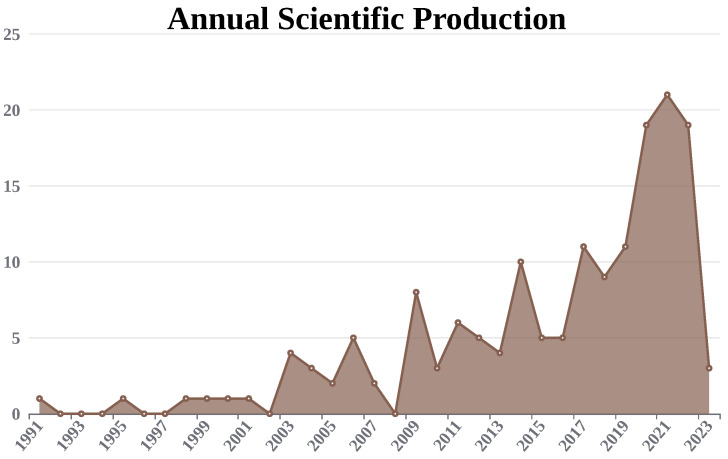
Annual Scientific Production.

Annual Scientific Production is an essential indicator for understanding the breakthrough or research trends in the field of study. Figure 2 shows the number of survey research articles published yearly in the umbilical cord, mesenchymal stem cell, and cryopreservation fields. From 2017 onwards, there has been a steady increase in survey article publications, which clearly indicates a sudden rise in research work in the field of study. This fact motivated us to present a review article focused on the ethical concerns associated with multipotent stem cells, banking, and cryopreservation. Increased research interest can be observed from 2003 itself, as shown in Figure 2.

**Figure 3:**
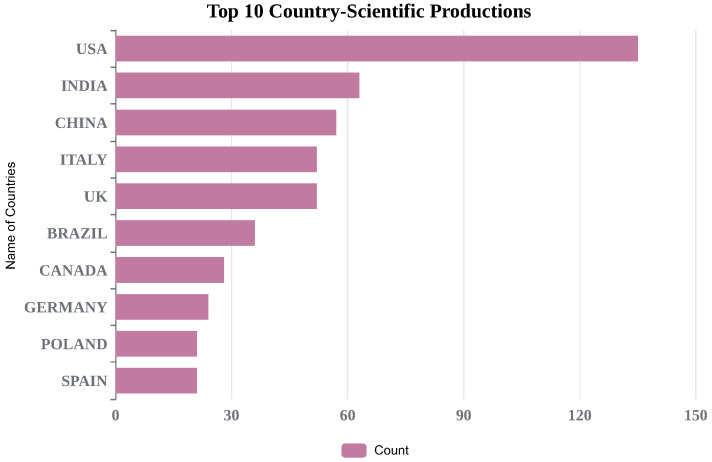
Country Scientific Production.

**Figure 4:**
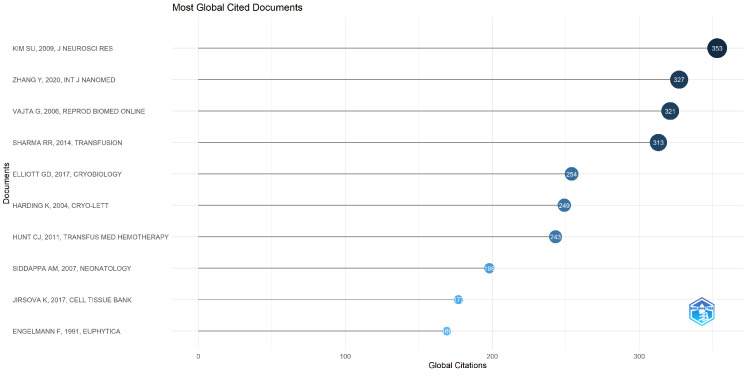
Most Commonly Cited Documents Globally.

**Figure 5:**
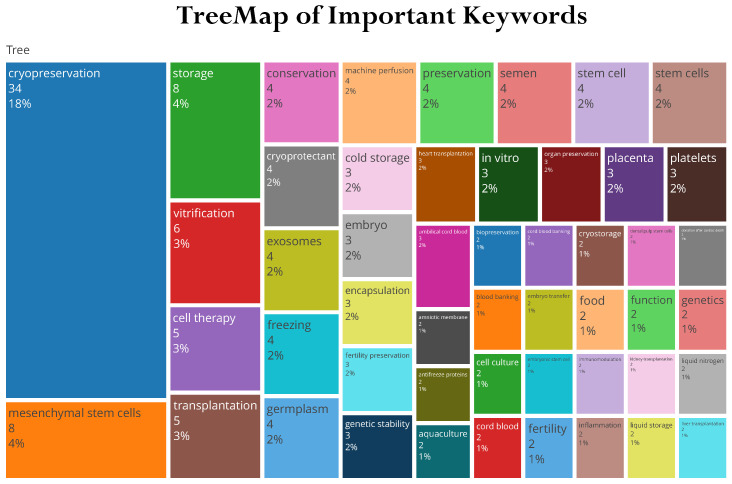
TreeMap Visualization.

**Figure 6:**
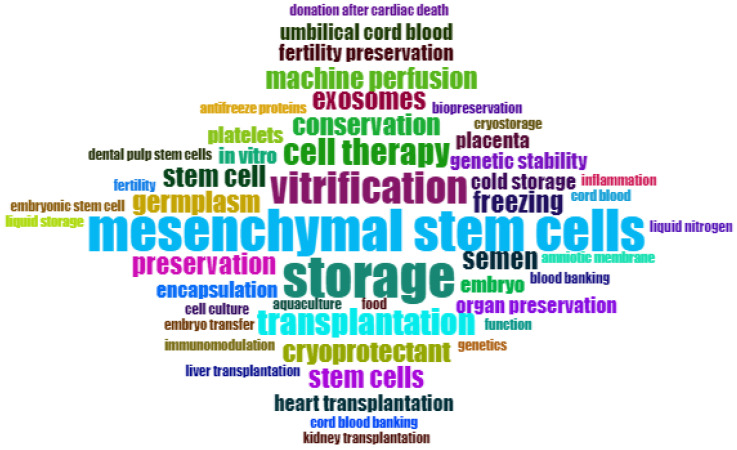
Important Author Keywords Visualization.

To better understand global-level research outcomes, country-level production is shown in Figure 3. India stands in the second position, with 63 research articles, and the USA is in the first position with 135 survey articles in the field of study. We have also included the most frequently cited articles in Figure 4. Additionally, Figure 5 and Figure 6 display important author keywords that are utilized by the majority of researchers, as represented through treemap and wordart visualizations.

### 2.3. Salient Contribution of the Proposed Survey Article

Most of the recent research has focused on explaining MSCs as a regenerative and repairable therapy in treating various disease conditions. Some researchers have worked on a mechanism for extracting exosomes from MSCs using nanomedicine vesicles. The current study briefly explained the current use of UCB stem cells to treat various diseases in adults and children. Ethical issues confronting UCB banking and its current availability in the various regions were reviewed. Table 1 tabulates the shortfalls of existing survey articles, which were motivated to propose this survey article.

## 3. UCB as a Regenerative Medicine

### 3.1. Stem Cell Transplants

Currently, hematopoietic cell transplants using stem cells from the UCB are deployed across the globe to treat cancerous and non-cancerous diseases [10]. A significant outcome from transplant procedures using this stem cell source is seen in treating different disease conditions, including hematologic, immunologic, malignant, and inherited metabolic disorders [10]. Blood stem cell transplants are required therapeutically to restore the body’s ability to produce blood and immune cells [38]. Hematopoiesis, or the development of hematopoietic stem cells, can form three types of blood cells: white, red, and platelets [39]. Several studies have found that UCB is an exceptional source of ingenuous cells for creating positive pluripotent cells. The composition of umbilical cord blood is known to be 40% monocytes and 40% lymphocytes, and the remaining 20% are neutrophils and progenitor cells [40]. Recent studies have shown that UCB is a rich source of CD34+ stem cells, although it has an inadequate cell dose and takes longer to engraft. In vitro, CD34+ cells of UCB proliferate at a higher rate than other bone marrow stem cells. When UCB is transplanted in vivo, its ability to regulate is enhanced as compared to bone marrow stem cells [40,41]. An expansion of adaptive immunotherapy employing other components of UCB, including regulating T cells, virus-specific T cells, and likely destroyer cells, has revolutionized the field and has enhanced the usefulness of UCB units [10].

According to current world count statistics (24 November 2022), more than 140 million babies are born annually. Thus, UCB is an abundant reservoir of regenerative cells waiting to be tapped [42]. As compared to other donor cell resources, the UCB collection is a safe, painless procedure, and it has a longer period of cryopreservation without affecting the characteristics of its viability and structure. It also has a reduced possibility of spreading viral infections and somatic mutations that can increase morbidity following a course of transplantation [43]. Furthermore, with the use of UCB, allogenic transplants are easily possible. It is estimated that over 115,000 solid organ transplants have been completed using UCB. Depending on the immunogenicity, some UCB cell populations verified intrinsic ‘immune privileged’ properties in reaction to interferon-gamma, by expressing the antigens class I and II HLA. It causes a reduction in UCB immunogenicity due to immaturity. As a result, specific UCB-derived cell lineages are valuable tools in modern regenerative medicine [44].

### 3.2. Ex Vivo Modulation Strategies to Enhance the Therapeutic Potential of UCB

Human Cord Blood (HCB) is intrinsically characterized by a low hematopoietic stem cell (HSC) count, which is related to deferred time engraftment, increased graft failure rates, and initial impermanence. Table 2 shows the number of HCTs performed and reported in reported in CIBMTR (2016–2020). Initially, it was hypothesized that 16-dimethyl prostaglandin E2 (dmPGE2) regulates hematopoietic stem cell homeostasis and has a short-lived ex vivo variation that could advance clients’ outcomes by progressing the efficiency of HSC dosage. According to the findings of North et al., elements that increase prostaglandin synthesis can increase HSC numbers. Cyclooxygenases promote PGE2 synthesis, which helps in the formation of HSC [45]. Molecular profiling approaches advanced HSC formation in clinical settings by determining ex vivo modulation settings such as temperature, time, concentration, and media. A phase I clinical trial is required to estimate the protection and probability of modulating a single UCB unit with dmPGE2 (ProHema) before abridged concentration binary UCB transplantation [46].

Table 2 illustrates that, despite umbilical cord blood being a rich source of stem cells that can effectively treat various illnesses, the rates of UCB transplantation remain lower when compared to other types of transplants.

### 3.3. Evolutions in the Use of Umbilical Cord Mesenchymal Stem Cells (MSCs)

Regenerative medicine uses pluripotent stem cell isolation and identification in clinical settings. Fresh UCB contains non-hematopoietic stem cells, endothelial cells, MSCs, and an unlimited number of somatic cells [47]. Currently, UCB stem cells are widely used to treat various cardiovascular, hepatic, ophthalmic, orthopedic, neurological, and endocrine disorders [10,48]. In 2006, the International Society for Cellular Therapy defined MSCs, and this was accepted by most researchers. The criteria are as follows: (a) MSCs are required to be plastic-free during conserved traditional culture settings, (b) MSCs induce endoglin-1, ecto-5′-nucleotidase, and thymocyte antigen-1 without CD45, integrin- M, or CD79-alpha [49]. Mesenchymal stem cells have the ability to self-renew and to differentiate in multi-lineage directions [50,51]. The major known sources of mesenchymal stem cells include bone marrow, umbilical cord tissue, Wharton’s jelly of the umbilical cord, UC tissue, amniotic fluid, and adipose tissue (MSCs) [52].

**Figure 7:**
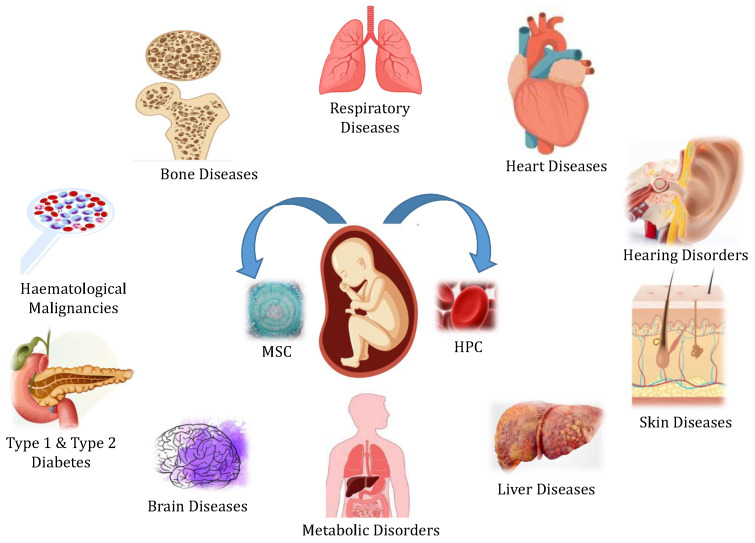
Use of UCB stem cells in various disease conditions—UCB stem cells are currently used in a variety of clinical applications.

Figure 7 shows different applications of UCB. It is a readily available blood resource for regenerative stem cells such as hematopoietic progenitor cells (HPCs) and MSCs to treat various human diseases. Promising outcomes have been observed in the treatment of brain injuries in infants and young children through the utilization of stem cells derived from UCB [53,54]. According to Dr. Harris’ report, the density within the vascular system of the heart increased in the animals treated with umbilical cord blood versus untreated animals [55]. Improvements in neurologic function after being injected with UCB stem cells was also noted in animal studies. When mice with weakened immune systems were given human cord blood-derived CD34(+) cells through their entire bodies, two days after experiencing a stroke, it led to the creation of new blood vessels in the area that lacked oxygen and nutrients due to the stroke. This also resulted in a positive setting for the regrowth of nerve cells [56].

Recent studies have stated that MSC-derived exosomes are easily manipulated and applied to be used in therapeutic regimens for the treatment of different disease conditions [57]. Current research shows that mesenchymal stem cells obtained from umbilical cord blood play a crucial role in wound healing, and that they produce exosomes to promote various signaling pathways that are advantageous for repairing damaged tissues, and the development of new cells [58]. Some of the case studies associated with the treatment of different ailments with UCB are summarized in Table 3.

UCB stem cells are one of the potential future therapeutic options for treating optical related diseases [59]. A study enrolled 68 patients with haematological malignancies who were administered myeloablative acclimatizing therapy. They were disease-free after forty months of umbilical cord blood transplantation [60]. The administration of mesenchymal stem cells has been shown to cause a reduction in inflammation in the respiratory system by suppressing transforming growth factor-β, interferon-γ, and pro-inflammatory cytokines [61]. Patients with bone disorders, such as orthogenetic disorders, benefited from treatment with umbilical cord mesenchymal stem cells [62]. Mesenchymal stem cell administration has shown improved an auditory brainstem response and distortion product otoacoustic emissions in children with hearing disorders. Additionally, promising results have shown a reduction in wound size by more than 80% [63,64]. Major clinical trials using various UCB stem cell products to treat different human diseases registered at www.clinicaltrials.gov are tabulated in Table 4.

**Table 3:** Summary of the case studies conducted recently.

Case Study Number	Condition	Sample Size	Notable Findings	Reference
1.	Post-COVID-19 complications—pulmonary edema	1	UCB mesenchymal cells reduce inflammation, including cytokine storms in COVID-19 patients, as indicated by decreased IL-4, IL-6, and IL-10 levels.	[65]
2.	COVID-19 with acute respiratory distress syndrome	24	Transfusing UCB stem cells safely increased patient survival to 91% (*p* = 0.015), and significantly improved recovery rates (0.03) by reducing cytokine storms.	[66]
3.	Autism spectrum disorder (ASD)	20	Administering UCB mesenchymal stem cells was safe and effective in improving ASD.	[67]
4.	Psoriasis	7	UCB MSC infusion had no side effects in 6 months of follow-up, increased Tregs and CD4+ memory T cells, and effectively treated 25% (2/8) and 66.7% (6/9) of male and female patients.	[68]
5.	Neurological disorders	100	UCB MSC administration for neurological disorders was safe and effective, with minimal side effects noted in one-year patient follow-up.	[69]
6.	Patients with muscular dystrophies	22	Two courses of 2–5 intravenous injections every two months significantly improved limb strength, stretching, bending, straightening, and gait (27.3%).	[70]
7.	Rats with skin wounds	-	UCB-MSCs assist in regenerating skin appendages, nerves, and arteries, aiding wound closure and controlling collagen distribution.	[71]
8.	Patients with alopecia lesions	3	No change was observed until day 53, and thereafter, minimal hair growth changes were detected in the first sample. The second patient showed significant hair growth on day 117 after transplantation By day 226, the third patient’s lesions had reduced, and they demonstrated remarkable hair growth.	[72]
9.	Acute Myeloid Leukemia (AML)	2963	Allo-HCT from an unrelated donor produces superior results to cord blood transplantation.	[73]

## 4. Collection and Cryopreservation of UCB

### 4.1. Umbilical Cord Blood Collection

Umbilical cord blood is replete with stem cell sources; thus, these cells are widely used to treat genetic disorders, blood cancers, and immunological disorders. UCB stem cells are also used to treat relatives and siblings who have immunological or other non-immunological diseases [74]. Before collecting blood from the umbilical cord, midwives/doctors must obtain informed consent from the donors. Mothers must be tested for biological infectious diseases such as HIV reactivity, and Hepatitis B and Hepatitis C. Furthermore, blood is tested for sterility using bioMérieux (Hazelwood, MO) [75]. In most cases, the blood from the cord is collected immediately after the newborn’s birth and before the placenta is expelled [76]. The first clamp needs to be applied just near the placenta, and the second clamp is applied at least 5 cm away from the baby’s umbilicus. The umbilical cord is smeared with spirit or betadine before blood collection to ensure sterility.

Umbilical cord blood can be collected either via syringe or bag systems. In the case of the syringe system, UCB is collected using syringes of various sizes, such as a 60 cc large syringe and a 200 mL small-sized bag. Experienced collectors usually collect blood with a syringe while maintaining high sterility. Blood collection for these collectors could take up to 5 min before the placenta is expelled. According to Burton’s theory, an accurate timing of cord clamping and the withdrawal of UCB is critical since umbilical blood vessels are prone to collapse due to the interruption of blood flow caused by pressure. Furthermore, this procedure is simple, noninvasive, and painless. The blood is drained into a sterile container after a simple venipuncture procedure. Nonetheless, the chances of UCB contamination are high during this simple procedure. Therefore, sterile techniques must be used when collecting umbilical cord blood [77,78,79]. Usually, neither the mother nor the baby experience risk during the collection of the blood samples as it is collected after the clamping of the cord. The collection of cord blood or cord tissue is considered to be safe for both vaginal and cesarean deliveries.

Bag collection is used by most collectors because, in their opinion, bag collection is easier than syringe collection as this procedure will be completed in two minutes. However, in both types of blood collection, sterile kits are pre-anticoagulated. They must be available with all of the necessary transportation materials, such as a double-layered restraint and a crush-resistant container. These kits must also have an optimal temperature of pH, carbon dioxide, and oxygen levels. Optimal temperature maintenance is necessary for transportation and storage prior to sending for processing, since this may have a substantial effect on cell viability, and several research papers had mentioned different ranges of temperatures for storage and shipping of the Umbilical Cord Blood Units (CBUs) such as 4 °C [80,81], 4 °C–7 °C [82], 4 °C–10 °C [83], and 4 °C–24 °C [84]. Additionally, there are debates about transporting CBUs at temperatures ranging from 15 °C to 25 °C [78]. The clotting of the entombed UCB delays the withdrawal of uncoagulated blood, which is the immediate significance of vascular obstruction. Clotting is one of the most challenging obstacles to optimal sample withdrawal. Furthermore, these kits are adequately shielded and padded with soft material to facilitate safe and secure transport by preventing physical damage. This facility also aids in temperature maintenance during transportation [77,78,85].

Before UCB collection, blood samples must be obtained from the mother for infectious disease marker (IDM) testing, which is a regulatory requirement, using the provided vacutainers. The blood drawn from the umbilicus must be transported to the lab within 28 to 34 h. The shipping process to laboratories can be successful within meticulous and recorded conditions. Preferably, a large number of UCBs should be processed partially via automation. Red Blood Cells (RBCs) are primarily depleted from UCB before cryopreservation [77,78]. Since the majority of stem cells reside in the mononuclear cell (MNC) fraction, which is only required for banking, this procedure ensures a greater number of stem cell retrievals, as red blood cells account for more than 50% of the blood collection. Furthermore, volume reduction benefits the UCB banks by reducing storage space and allowing for a reduction in dimethyl sulfoxide (DMSO) quantities in cellular products; it also reduces cytotoxicity caused by RBC defrosting [86,87,88]. Multiple procedures are used to improve the viability of stem cells, including density gradient separation and gelatin sedimentation [32].

**Figure 8:**
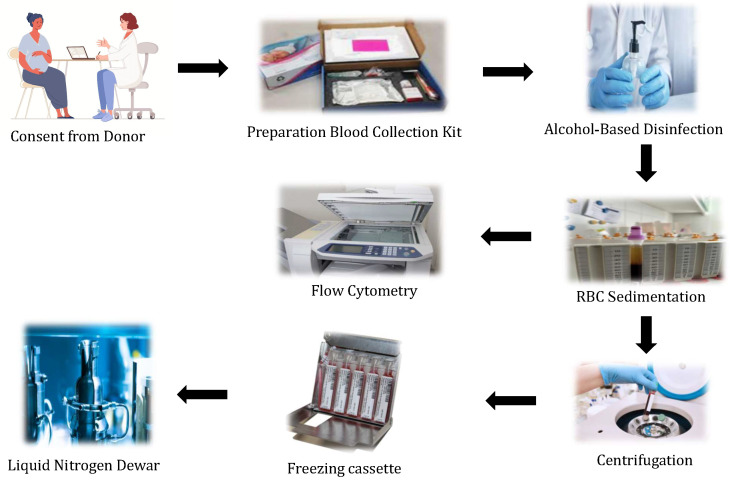
Process of UCB collection, cryopreservation, and banking.

The UCB must be padded and insulated to regulate the temperature after being collected in the kit (Figure 8). Before entering the main laboratory, the external surfaces of the UCB bags must be disinfected with an alcohol-based solution. After RBC sedimentation with hydroxyethyl starch and centrifugation, a pre-cryopreserved cell deferment is enhanced with mononuclear cells under strict aseptic conditions. The quality of cryopreserved UCB is routinely expressed based on the estimated total nucleated cells (TNCs), the evaluation of CD34+ and CD45+ cells, and cell variability.

### 4.2. Cryopreservation of Umbilical Cord Blood

In cryopreservation, very low temperatures are used to maintain the structural and functional integrity of cells and tissues; in this period, the aqueous phase classically endures the ice formation phase. Once frozen, the cells and tissues can be stored in a stable state as the low sub-zero temperature achieved is optimal, which is typically at or near the temperature of liquid nitrogen (−196 °C) [89]. Cryopreservative agents must be used for the cell’s survival and to maintain the structural integrity of the cells. An alternative procedure to cryopreservation, is vitrification, in which a solidification of the aqueous system occurs, without the crystallization and growth of ice. Umbilical cord blood is cryopreserved using an automated microprocessor-controlled cell freezer. Over 20 min, an equivalent amount of the cryoprotectant dimethyl sulfoxide (DMSO) is gently and slowly added to the autologous plasma. According to the cryopreservation protocol, the temperature is gradually reduced to −80 °C under the control of a controlled-rate freezing process. The cells are stored in a specially constructed liquid nitrogen freezer (MVE, Inc., Laguna Beach, CA, USA) at the end of the freezing procedure, which allows for vapor storage at liquid nitrogen temperatures [90,91]. The use of autologous plasma in cryopreservation is crucial for preventing cell exposure to non-self and animal proteins. To maintain a regulated and recorded cryopreservation run for each and every frozen sample, appropriate temperature protocols governing the freezer must be used in accordance with the regulatory bodies’ guidelines. Distinctive procedures for UCB cryopreservation have already been implemented over time. Similarly, liquid- or vapor-phase nitrogen is used to store and process the UCB, in order to conserve the practicality and probability of the cell artifact [77,92].

As per the results of Broxmeyer et al. in [22], UCB preservation has had no significant impact on cell viability and production over the last 20 years. Furthermore, industrialized small-scale automated cryopreservation systems such as the Mini-BioArchive system (Cesca Therapeutics Inc., Rancho Cordova, CA, USA) have provided sufficient cellular products for UCB transplantation [93]. Technically, the banking of UCB uses two kinds of freeze cell products: plasma depletion (PD) or red cell reduction (RCR). The PD techniques remove the plasma cells and hoard all cells, freezing them in 10% dimethyl sulfoxide (DMSO). In the RCR technique, UCB is centrifuged in albumin solution to separate 21 mL of UCB, the majority of which is WBC; four ml of 50% DMSO are added, resulting in 25 mL of frozen cell suspension [94]. Although UCB methodologies are less costly to process, they are costlier to the bank and more difficult to defrost. Even though optimally washed and defrosted platelet-depleted UCB units have more total nucleated cells (TNCs), CD34+ cells, colony-forming units, and advanced cell engraftment amounts than red blood cells; they are used to treat diseases such as thalassemia [92,95].

The addition of DMSO to the UCB before freezing aids in cell protection by controlling the formation of intracellular ice crystals. However, more than 1% of DMSO can harm blood cells for more than 30 min at 37 °C. As a result, DMSO must be removed after defrosting to reduce adverse effects on transplanted patients [94]. Only about 10% of cord blood banking units have enough cells for use in adult transplants. Barker et al. reported positive results, with approximately 23 recipients receiving a binary partially human leukocyte antigen (HLA), comparable to UCB units [96,97].

## 5. Public and Private Umbilical Cord Blood Banking

The New York Blood Center opened the first UCB bank in 1993. In Asia, Europe, Oceania, North America, and South America, there are a total of 100 UCB banks. Around 5 million UCB units are in use across the world. A total of 800,000 UCB units are owned by public banks, while private banks own the rest. Across the world, around 35,000 UCBTs were performed till 2019, whereas the United States alone performed 2803 from 2016 to 2020. Under ideal conditions, the biological qualities of UCB units can last for more than 10–20 years. Because UCB is readily available from unrelated donors, the search time is shortened from three to four months for bone marrow and two weeks for peripheral blood [78].

Private banks are profit organizations that provide services to store UCBs for individual use. These organizations incur storage fees but do not guarantee that the UCB from a person is useful to treat a specific disease condition of the same person (autologous UCB transfusion) or a family member [98]. The fundamental goal of private banks is to retain the high-quality UCB units used to transplant hematopoietic stem cells as regenerative medicine. Several technologies have emerged to increase the number of HSCs and to speed up engraftment. At the same time, the public UCB banks provide free services to store blood for individuals who meet the requirements for donation. These banks are typically supported or funded by federal or private companies, allowing them to provide free collection and storage services. Personal engaged storage is not permitted in public UCB banks. The UCB in public banks is available for all patients to transplant, which is known as allogenic UCB transfusion, and it is not limited to a family or an individual [98].

The benefits and drawbacks of public versus private UCB banks vary, depending on the patient’s needs, which may differ. The concept of autologous and allogenic UCB should be discussed. Because stored blood contains the same genetic material, UCB stem cells collected from newborns cannot be used to treat cancers or other genetic disorders in the same individual. However, researchers and healthcare professionals must provide accurate information to parents about the goals and indications of UCB banking, as this is one of the most important ethical concerns [99]. Public UCB banking is widely recommended for obtaining umbilical cord blood for transplantation, therapeutic use, and other medically validated indications. Public banks typically encourage allogenic donations, which are similar to blood collection in various blood banks [98].

The following are the public cord banks in India.

1. Jeevan Stem Cell Bank was founded in 1995 and is supported by the Tamil Nadu government. These banks primarily treat leukemia, thalassemia, and other hematological disorders.
2. The Reliance Dhirubhai Ambani Life Sciences Center in Thane, Maharashtra, is supported by Reliance Life Sciences Pvt. Ltd. Free UCB; collection and storage services are provided.
3. The School of Tropical Medicine (STM) established Kolkata’s first public cord blood bank.
4. StemCyte Inc., Apollo Hospital Enterprises Ltd., and Cadila Pharmaceuticals Ltd. founded StemCyte India. The bank provides collection, processing, testing, and storage services for private and public umbilical cord blood units, and therapeutic applications.
5. LifeCell was established in 2004 in technological collaboration with cryo-cell international. The primary goal of this bank was to assist patients in receiving lifesaving stem cell transplants to increase their chances of receiving the same stem cells.

## 6. Ethical Concerns in UCB Banking

Legal aspects and regulatory norms are of prime importance in the practice of UCB banking. They include obtaining informed consent, medical suggestions, proprietorship, entitlements connection to medical welfare, uses pertaining to allogenic versus autologous, legal outlines, public and private banks, budgetary systems, entrée and society, quality assurance, tracing and tracking systems, associated cost, publicizing, patenting, the protection of individual data, and maintaining confidentiality; and relations between receivers, patients, physicians, and UCB banks. Another emerging issue is the debate on donations and self-preservation at UCB banks. To address this issue, various governmental and non-government bodies have drafted rules and regulations to meet and resolve maximum concerns [37,100]. An additional apprehension with UCB is that it should not alter routine obstetric or newborn care practices, such as delayed umbilical cord cutting and clamping, except for some of the rarest medical conditions. The time of clamping and cutting the cord became a point of disagreement for UCB collection for some obstetricians [37]. Table 5 shows the common challenges associated with UCB banking.

### Recommendations of Professional Organizations Regarding UCB

According to the American College of Obstetricians and Gynecologists (ACOG), pregnant women should be given unbiased information about UCB. According to ACOG statistics, the estimated need for UCB transplantation to a baby or a relative is about 1 in 2700. As a result, ACOG recommends UCB only if a family member has a history of or is currently undergoing stem cell treatment, and not for future anticipated uses. According to the American Academy of Pediatrics (AAP), UCB should be regarded as free biologic insurance. It also observes that many private UCB banks are unfounded. It advises storing UCB only in public banks for general public use. The AAP recommends private UCB only if any family members are currently receiving stem cell treatment or if a diagnosis for stem cell treatment is required. Lamaze International (2010) also prohibits the advertising of cord blood collection practices, particularly by private UCB banks.

## 7. Discussion

Clinical trials addressing MSC-based interferences in potential regenerative medicine applications are gradually expanding globally. Because UCB has a rich supply of MSCs that can be acquired non-invasively from the placenta or umbilicus, many UCB banks are establishing numerous programs to collect and store UCBs. UCB is likely to provide one of the most clinically successful and therapeutically driven MSC-based therapies. The present study involves discussions on the benefits of using mesenchymal stem cells derived from Umbilical Cord Blood (UCB) in various disease conditions, as evidenced by recent research. It also covers the biology and functionality of the cryopreservation of UCB cells, and the crucial role played by midwives and obstetricians working in labor rooms in the collection process. Additionally, the study examines the hospital standard operating protocols for the collection of UCB and the ethical concerns surrounding UCB banks.

### 7.1. UCB-Derived Mesenchymal Stem Cells: Benefits and Usage in Recent Research on Various Disease Conditions

The benefits of UCB-derived mesenchymal stem cells are diverse, and its use in treating a variety of medical situations has yielded encouraging results. For more than three decades, researchers have conducted unpackaged clinical trials to assess its operation and outcome in diverse illnesses. In more recent applications, for COVID-19 patients with pulmonary edema, UCB MSCs appeared to reduce disease inflammation and cytokine squalls [65,66]. Even children with autism spectrum disorder (ASD) have been shown to be effectively treated with UCB-derived MSCs [67]. Regulator T cells (Tregs) and CD4+ memory T cells increased quickly in 25–66.7% of female and male psoriasis patients. Furthermore, patients with neurological problems demonstrated significant benefits [69]. However, the presence of CD4+ and CD25+ T cells, and Foxp3 in umbilical cord blood and peripheral blood need more clarity, with various authors presenting differing claims [101,102,103]. Similarly, individuals with muscular dystrophies improved significantly in limb strengthening, stretching, bending, and straightening, with 27.3% improving their gait as well [70]. Three alopecia patients responded well by sprouting their hair from the 53rd to the 226th day of treatment. It is pertinent to note that UCB stem cells are currently routinely used in haematological illnesses, cancerous and non-cancerous, metabolic, and congenital diseases [72].

### 7.2. Cryopreservation of UCB Cells: Understanding Their Biology and Functionality

Based on clinical experiments on the cell biology and functionality of the cryopreservation of UCB cells, UCB from a single donor was used as a resource for MHC-matched allogenic transplantable hematopoietic repopulating cells. According to current cryopreservation protocols, there is no need to wash the cells before freezing or otherwise handling them before defrosting, as these treatments can cause the loss of hematopoietic cells [74]. Thus, the following strategies vastly increase the availability of UCB stem cells in an emergency transfusion. Furthermore, UCB can be conveniently collected at the time of birth without putting the other person or the baby at risk. These theoretical inklings encourage investigators to conduct additional clinical trials, as UCB represents a potential source of transplantable hematopoietic stem cells.

Umbilical cord blood has one log less total nucleated cells (TNCs) and CD34+ cells than BM or PB, resulting in delayed engraftment and greater graft failure rates [19,23]. According to the findings of previous studies, UCB contain a greater number of HPCs and multipotent colony forming cells than PB and BM CD34+ cells. Additionally, CD34+cell progenitors in umbilical cord blood proliferated rapidly and have the ability for multiple cell division [40,104,105]. Furthermore, in vitro and in vivo data show that the proliferation and development rates of HPCs are too high, which could be attributed to UCB cells exiting in G0 and G1 quicker than adult bone marrow progenitors. As a result, the patients’ UCBT frequencies and IL-10-producing Bregs number heal faster [106]. Despite the fact that UCB is easily accessible and associated with lower rates of graft versus host disease (GVHD), insufficient quantities of whole nucleated cells and CD34+ cell dosage forms in UCB units cause delayed hematopoietic healing and increased rates of graft failure, which tend to increase the infection rate. To address these obstacles, scientists are investigating various methodologies to improve the effectiveness of UCB transplants.

### 7.3. Roles of Midwives and Obstetricians in UCB Collection

Midwives and obstetricians working in labor rooms or birthing centers must advise the prenatal woman and her husband about the UCB-generated MSCs and the benefits of storing them in public banks. Parents are encouraged to express their views and interests in donating UCB for storage; however, they must communicate their decisions at least two months before to the delivery date, as all hospitals may not routinely collect the UCB for public banking. The collection and storage of UCB by public UCB banks is free of charge. The public cord banks linked with the hospital must be contacted to determine the eligibility for donating UCB. By taking a complete history, the donor should be checked for infectious agents such as HIV and Hepatitis B. When a mother meets the qualifying conditions, she is asked to sign a consent form. If a family wishes to store the UCB for the use of their family members, they are advised to create a contract with a private bank in which the family members must pay an initial charge, and the yearly preservation fee varies from bank to bank. However, the donation of UCB in a private bank is still controversial. Many obstetricians and prominent organizations, including the American College of Obstetricians and Gynecologists (ACOG), the American Academy of Paediatrics (ACP), and the American Society of Bone Marrow Transplantation (ASBMT), and American Academy of Pediatrics Policy Statement do not recommend donors to donate and store in private banks unless there is a demand within the family for which the UCB may be banked [107,108,109,110]. A study found that around 80% of obstetricians were interested in discussing UCB banking alternatives, but 49% of patients were uninformed of the UCB [111]. However, further research is required to comprehend parents’ curiosity about UCB donation in private or public banks, and a geographical study will aid in determining the opinions of healthcare providers and obstetricians regarding UCB storage, and the ethical considerations associated with UCB donation. Essentially, child birth educators must be well-versed in facts about UCB collection and storage in order to effectively handle queries raised by parents.

### 7.4. Hospital Protocols for the Safe and Efficient Collection of UCB

Based on individual hospital standard operating protocols, UCB collection begins just after the infant is delivered, and either before or after the placenta is delivered. The UCB is collected in a sterilized bag, which has to be labeled and preserved at the proper temperature before being sent to the bank. Again, various studies provide varying storage and transport temperatures. However, the typical temperature maintained while transferring blood to storage is 15 °C to 25 °C [78]. The UCB blood will be transported to a bank for examination, freezing, and storage. The unit determines if the bag seems to have enough blood-forming cells. The blood is frozen and kept at a low temperature for later use. For the past 15 years, the typical cryopreservation temperatures have been −196, −156, or −80 °C, reflecting the storing temperatures in liquid- and vapor-phase nitrogen, and in cryopreservation automated freezers, respectively. The current temperature range for cryopreserving the UCB is −196 to −80 °C [92,112,113]. The duration of cryopreservation is still unknown; many studies have proposed varying times. One recent study found that the average range of the UCB cryopreservation period is between 0.7 and 13.4 years. An average of 10 years is considered safe for utilizng the UCB derivatives. There was no significant difference between cryopreservation as well as the potential of neutrophil engraftment, in their investigation.

### 7.5. UCB Banks: Addressing Ethical Concerns in Establishment and Operation

Ethical concerns are a major source of contention in preserving and collecting umbilical cord blood. Despite medical technological trends, there are still ethical concerns with UCB collection and storage. These concerns are connected to the topic of patients’ rights and involvement in medical decisions [114]. This challenge is one of the key requirements that must be reflected in the inclusive health system [37]. One of these difficulties is respect for the autonomy and independence of persons who can refuse or accept particular treatment. Many researchers in our current review have raised various concerns about whether cord blood should indeed be preserved, in either private or public institutions.

The samples are stored in private banks after the applicant submits an application and pays the initial expenses. The parties own the samples taken as per the contract [115,116,117]. Parents can contribute to the UCB for free in public banks, and the public banks then owns the cord blood samples. A critical concern is that family members should be treated fairly when it comes to obtaining banking facilities. However, studies have shown that using private banks has several difficulties, including a high economic cost. Public banking services are useful; however, the waitlist may be longer, putting people’s lives in jeopardy [118]. Another recurrent issue addressed in studies reviewed is the disclosure of the donor and recipient’s names, which are frequently published in government banks. Can the donor be told of the beneficiaries’ names? It is a frequently asked question. Banks are accountable for protecting the privacy of contributors and beneficiaries [119].

An important problem identified in the research regarding donating or storing the UCB is obtaining informed consent from the donor. However, obtaining patient consent before beginning any inquiry or therapy is a concept that has been introduced previously in the medical sector. Healthcare providers and banks must be aware of the measures involved in obtaining consent before engaging in any medical activity in the hospital. It will safeguard them and also keep their patients well-informed.

Unless the regulations regarding UCB banking and transplant are strong and contemporary, major concerns regarding the lack of practical knowledge and relevant information by healthcare workers, blatant advertising by private companies, the emotional lure of vulnerable parents for their babies’ future, the monetary aspects involved, the usefulness of UCB stored in private banks, the success/failure of cord banking and future availability, the social estrangement of a large, needy population due to a lack of knowledge and funds, and the ever progressing science of UCB transplants will continue to ail us.

In this study, the necessary information is extracted from publications available in the SCOPUS and PubMed databases. While many other databases contain relevant data, SCOPUS is widely used and provides an enormous amount of information. The research work can be extended by including data from other databases such as Google Scholar, Web of Science, Delnet, and Pearson. However, time constraints prevented their inclusion in this article. However, these databases are planned to be included in upcoming studies. It is important to note that this study is limited to publications published in the English language.

## 8. Conclusions

The scientific and therapeutic use of umbilical cord blood has evolved substantially over the last 30 years or so. Even though considerable issues remain concerning UCB collection, preservation, banking, and services, the meticulous and honest execution of the various policies proposed by healthcare policymakers can overcome these challenges and issues. According to the current reviewer, the UCB process and treatment should begin with counseling pregnant women about the UCB donation, and continue until the needy patients obtain stem cells. Some obstacles related to the UCB procedure can be mitigated by having well-informed healthcare workers working in prenatal and intranatal wards who are armed with adequate knowledge and skills about UCB collection, processing, and ethical considerations. Midwives or obstetricians should be able to provide precise information about the UCB process to parents during the antenatal period, enabling them to make a well-informed choice two months prior to delivery. The uniqueness of this study is that a significant amount of information relevant to the UCB process is easily accessible to healthcare professionals.

## Figures and Tables

**Table 1:** Significant contribution to research related to the proposed research survey.

Significant Contribution	Number of Papers Surveyed	Specific Study Topic	Short Falls	Ref.	Year
UBT use in Oman, and its benefits and drawbacks	29	UCB Stem Cell Transfer in Oman: Banking and Transplantation	Umbilical Cord Blood Banking and Stem Cell Transplantation	[27]	2011
Preservation of Cord Blood for autogenic/allogenic therapy	54	Adverse Effects of Transfusing Placental Red Blood Cells (RBCs) and Umbilical Cord Blood (UCB)	Clinical trial results and reviews must be accurately specified	[28]	2013
This study aids the clinician in deciding whether to bank the UCB	31	Current guidelines for cord blood unit selection have implications for transplantation and banking decisions, as reflected in graft adequacy measures	The source of stem cells, such as UCB, bone marrow, and placental products, can influence stem cell banking	[29]	2013
The future of UCB-derived cellular therapy	39	Clinical studies on umbilical cord blood-derived cell transplantation for regenerative therapy are the main focus	Advising on Changes in Banking Procedures for Umbilical Cord Blood (UCB) Stem Cells in Response to Emerging Cell Therapy Trends	[30]	2013
An overview of the procedures for banking cord blood	52	Transplanting multiple units or ex vivo transplantation can overcome the UCB transplant’s cell dose limitations	The abstract does not explain the accreditation guidelines for UCB banking quality	[31]	2014
This study thoroughly explains the current and potential clinical applications of UCB	94	Problems associated with UCB cryopreservation and processing	Umbilical cord MSCs in regenerative therapies	[32]	2015
Clinical trials in children using MSCs from various sources to treat diseases	158	MSC sources, therapies in clinical trials, route, dose, and timings	UCB MSC sources unclear, placental derivatives and amniotic fluid only treat 5 diseases	[33]	2016
This article guides clinicians on how to distinguish the MSCs from the UCB.	60	Current MSC separation techniques, their values, benefits, and constraints	----	[34]	2017
Improvements in mesenchymal stem cell (MSC) exosomes	95	Exosome characteristics and biological functions in clinical practice	Information about how to obtain MSC exosomes is insufficient	[35]	2021
Nanomedicine vesicles are utilized in both nanotherapeutics and as drug delivery systems	161	The use of nanomedicine vesicles in translational research—exosomes—to overcome limitations in cell-based therapies	Comprehensive comprehension of exosomes’ composition and mechanism are crucial for their proper clinical application, given the risk of off-target activity	[36]	2021
There is a call for policymakers and medical professionals to create clear ethical standards for UCB banks.	52	UCB banks’ ethical issues in collecting and preserving blood	Private and public banks need individual comparisons due to their unique challenges	[37]	2022

**Table 2:** Number of hematopoietic cell transplantations (HCTs) performed and reported in CIBMTR (2016–2020).

Donor Type	Cell Source	2016, No.	2016, Col %	2017, No.	2017, Col %	2018, No.	2018, Col %	2019, No.	2019, Col %	2020, No.	2020, Col %
Allogenic	Bone Marrow	2011	23	2071	23	2179	23	2014	21	1507	17
Allogenic	Cord Blood	682	8	621	7	557	6	512	5	422	5
Allogenic	Peripheral Blood	6065	69	6343	70	6580	71	6865	73	7097	79
Autologous	Bone Marrow	22	<1	35	<1	27	<1	23	<1	22	<1
Autologous	Cord Blood	0	0	0	0	4	<1	2	<1	1	<1
Autologous	Peripheral Blood	12,847	100	13,337	100	13,477	100	13,710	100	12,951	100

**Table 4:** Major clinical trials using UCB stem cell products to treat different human diseases, registered at www.clinicaltrials.gov (accessed on 24 May 2023).

Status	Category	Disease Conditions	Transplantation Type	Identifier	Participants	Phase
Completed	Metabolic disorders	Type 1 Diabetes	Autogenic	NCT00873925	23 children	1
Unknown		Type 1 Diabetes	Autogenic	NCT00989547	18	1
Recruiting		Type 2 Diabetes	Allogenic	NCT03835312	50	NA
Recruiting		Type 2 Diabetes	Allogenic	NCT04441658	30	1, 2
Recruiting		Type 2 Diabetes	Allogenic	NCT04501341	15	1, 2
Completed	Hematological Disorders	Sickle Cell Disease Transfusion Dependent Alpha- or Beta-Thalassemia	Allogenic	NCT02179359	25	NA
Recruiting	Hematological Malignancies	Acute Erythroid and Lymphoblastic Leukemia	Autologous or allogenic	NCT04083170	10	2
Recruiting		Leukemia, Myelomonocytic, Acute	Allogenic	NCT04687657	20	1
Recruiting		Severe Aplastic Anemia and Hypoplastic MDS	Allogenic	NCT03173937	37	1, 2
Recruiting	COVID-19 diseases	COVID-19 Infection	Allogenic	NCT04565665	70 participants	1, 2
Not yet recruiting		COVID-19-Associated ARDS	Allogenic	NCT05092724	20	NA
Recruiting		COVID, Pulmonary Infection, Sars-CoV2	Allogenic	NCT04457609	40	1
Suspended	Ear Disorders	Hearing Loss	Autologous	NCT01343394	10	1
Completed		Sensorineural Hearing Loss	Autologous	NCT02038972	11	1, 2
Recruiting	Heart Diseases	Congenital Heart Disease, SRV Dependent	Autologous	NCT04907526	30	1
Active, not recruiting		Congenital Heart Disease, SRV Dependent	Autologous	NCT03431480	1	1
Completed		Hypoplastic Left Heart Syndrome	Autologous	NCT01883076	30	1
Completed	Autism	Autism	Autologous	NCT01638819	30	2
Completed		Autism Spectrum Disorder	Allogenic	NCT04710810	30	1
Completed	Neurological disorders	Cerebral Palsy	Allogenic	NCT03826498	40	2
Completed		Dementia of the Alzheimer’s Type	Autogenic	NCT01696591	9	1
Completed		Stroke	Autogenic	NCT03004976	83	2
Recruiting		Spinal Cord Injuries	Autogenic	NCT03979742	18	2
Unknown		Ischemic Stroke and Cerebral Infarction	Autogenic	NCT01438593	6	1
Withdrawn		Arterial Ischemic Stroke (AIS) in Children	Autogenic	NCT01700166	0	1
Unknown		Neonatal Hypoxic- ischaemic Encephalopathy	Autogenic	NCT02881970	20	1, 2
Completed	Skin Diseases	Androgenic Alopecia	Allogenic	NCT03676400	84	NA
Unknown		Diabetic Foot and Critical Limb Ischemia	Allogenic	NCT01216865	50	1, 2
Terminated		Epidermolysis Bullosa	Autogenic	NCT00881556	3	Early Phase 1
Recruiting	Liver and GI Diseases	Primary Biliary Cirrhosis	Allogenic	NCT04522869	34	1, 2
Recruiting		Hepatitis B	Allogenic	NCT03826433	20	1
Recruiting		Liver Cirrhosis	Allogenic	NCT05331872	20	1
Recruiting		Neonatal Necrotizing Enterocolitis	Autogenic	NCT05138276	60	Early Phase 1
Recruiting		Growth and Development	Autogenic	NCT05138276	60	Early Phase 1
Recruiting		Preterm Infants	Autogenic	NCT05138276	60	Early Phase 1
Recruiting		Nutrition	Autogenic	NCT05138276	60	Early Phase 1
Recruiting		Ulcerative Colitis	Allogenic	NCT04882683	50	NA
Unknown		Crohn’s Disease	Allogenic	NCT02000362	24	1, 2
Completed	Bone disorders	Severe Osteopetrosis	Allogenic	NCT00775931	7	2, 3
Suspended		Osteonecrosis of Femoral Head	Allogenic	NCT03180463	30	1, 2
Recruiting		Congenital Bone Marrow Failure Syndromes	Allogenic	NCT01962415	100	2
Completed	Respiratory tract diseases	Bronchopulmonary Dysplasia	Allogenic	NCT02381366	12	1, 2
Completed			Allogenic	NCT01297205	9	1
Completed		Respiratory Tract Infections	Allogenic	NCT02023788	8	NA
Completed		Premature Birth of Newborn	Allogenic	NCT02023788	8	NA
Not yet recruiting		Severe Acute Respiratory Syndrome (SARS) Pneumonia	Allogenic	NCT04299152	20	2
Recruiting		COPD	Allogenic	NCT04433104	40	NA

**Table 5:** Common Recurring Challenges in Reviewed Studies.

Private or public ownership of the banks	Major investigations have been conducted to determine whether or not the owners of private banks work for only commercial interests. Another concern relates to the ownership of stem cells by commercial and public banks, the methods for obtaining services from these banks, and the design of justice for access to bank services. It has been observed that private banks work to increase their economic capital, while general banks assist those in need.
Informed consent	Fifteen articles mentioned the requirement of informed consent from the donors and receivers. Obtaining informed consent before doing any investigative or therapeutic actions on patients is a concept that has been introduced previously in the medical field. It is one of the most important foundational concepts in the medical industry, and it will result in favorable ethical and moral outcomes. It is one of the most critical aspects of the patient’s right to donate UCB to the banks. It will give patients a better understanding of UCB collection and storage advantages.
Justice to access the services	The UCB stem cell banks are obligated to provide equal assistance to the needy; they must not work solely for economic gain. Equality is a crucial criterion or indicator for determining the success of health-related challenges. One of the primary concerns is that everyone should benefit from these services, regardless of their social or economic status.
Conflicts of interest	Three studies in our review mentioned the conflict of interest involved in the umbilical cord blood banking process. Corruption is the primary source of conflicts of interest. Conflicts of interest must be avoided and managed. When an organization or center’s interests collide with the lives and health of individuals, the conflict of interest is highlighted.
Social Problems	People’s perspectives on health concerns are essential in determining the success of any endeavor. Many studies have found that people need to be properly informed about UCB banks and their services. Efforts must be made to promote the process of UCB banking and to improve people’s perceptions of it by offering training. This is so that people can access suitable services, and so that healthcare professionals can be motivated to expand UCB operations in the health sector. People in some countries do not have a positive attitude toward banking because they may have received little or wrong facts about UCB banking.
Confidentiality	The actions of confidentiality in the hospital are a fundamental entitlement for patients, that cannot be taken away from them. According to the findings of the three surveys, many people voiced concerns about the confidentiality of their personal information in such banks. Doubts were expressed that their cells may be employed for something else. Donors and recipients were also concerned that their personal information might be shared with someone else. According to certain studies, the bank’s operations should be transparent.

## Data Availability

Not applicable.

## Abbreviations

The following abbreviations are used in this manuscript:

AAP	American Academy of Pediatrics
ACOG	The American College of Obstetricians and Gynecologists
ASBMT	American Society of Bone Marrow Transplantation
ARDS	Acute respiratory distress syndrome
COPD	Chronic Obstructive Pulmonary Disease
CBUs	Cord Blood Units
CIBMTR	Center for International Blood and Bone Marrow Transplant Research
dmPGE2	Dimethyl prostaglandin E2
dUC	Differential Ultracentrifugation
DMSO	Dimethyl sulfoxide
ELISA	enzyme-linked immunosorbent assay
HCB	Human Cord Blood
HCT	Hematopoietic cell transplantation
HPCs	Hematopoietic progenitor cells
HLA	Human leukocyte antigen
HSCs	Hematopoietic stem cells
HIV	Human Immunodeficiency Virus
IDM	Infectious disease marker
MNC	Mononuclear cell
MSC	Mesenchymal stem cells
MDSs	Myelodysplastic Syndromes
nPES	Nanoplasmon-Enhanced Scattering
RBCs	Red blood cells
TNCs	Total Nucleated Cells
UCB	Umbilical Cord Blood
